# Persons Claiming to Be Graduates of Ohio College Den. Sur.

**Published:** 1852-01

**Authors:** 


Persons Claiming to be Graduates and Students of the Ohio College of Dental Surgery.—We hear of many such whose names are not on the books of the institution, some of whom we feel assured never saw the College, and are not known by any member of the faculty. We would remind the profession and the public, that our graduates receive regular diplomas, and all the students, tickets from each of the professors. These should be taken as evidence of such claim, but not the mere assertion of an irresponsible individual. Those having honorary degrees itself, should always be able to show their diplomas.

